# Mitochondrial DNA mutation exacerbates female reproductive aging via impairment of the NADH/NAD^+^ redox

**DOI:** 10.1111/acel.13206

**Published:** 2020-08-03

**Authors:** Liang Yang, Xiaobing Lin, Haite Tang, Yuting Fan, Sheng Zeng, Lei Jia, Yukun Li, Yanan Shi, Shujing He, Hao Wang, Zhijuan Hu, Xiao Gong, Xiaoyan Liang, Yi Yang, Xingguo Liu

**Affiliations:** ^1^ CAS Key Laboratory of Regenerative Biology Joint School of Life Sciences Hefei Institute of Stem Cell and Regenerative Medicine Guangzhou Institutes of Biomedicine and Health Chinese Academy of Sciences Guangzhou Medical University Guangzhou China; ^2^ Guangzhou Regenerative Medicine and Health Guangdong Laboratory Guangdong Provincial Key Laboratory of Stem Cell and Regenerative Medicine South China Institute for Stem Cell Biology and Regenerative Medicine Institute for Stem Cell and Regeneration Guangzhou Institutes of Biomedicine and Health, Chinese Academy of Sciences, Guangzhou, China; University of Chinese Academy of Sciences Beijing China; ^3^ The Sixth Affiliated Hospital of Sun Yat‐sen University Guangzhou China; ^4^ State Key Laboratory of Respiratory Disease Guangzhou Regenerative Medicine and Health Guangdong Laboratory Guangzhou Institutes of Biomedicine and Health Chinese Academy of Sciences Guangzhou China; ^5^ Department of Epidemiology and Biostatistics School of Public Health Guangdong Pharmaceutical University Guangzhou China; ^6^ Synthetic Biology and Biotechnology Laboratory State Key Laboratory of Bioreactor Engineering Shanghai Collaborative Innovation Center for Biomanufacturing Technology East China University of Science and Technology Shanghai China

**Keywords:** aging, fertility, mitochondria, mitochondrial DNA, nicotinamide mononucleotide

## Abstract

Mammals' aging is correlated with the accumulation of somatic heteroplasmic mitochondrial DNA (mtDNA) mutations. Whether and how aging accumulated mtDNA mutations modulate fertility remains unknown. Here, we analyzed oocyte quality of young (≤30 years old) and elder (≥38 years old) female patients and show the elder group had lower blastocyst formation rate and more mtDNA point mutations in oocytes. To test the causal role of mtDNA point mutations on infertility, we used polymerase gamma (POLG) mutator mice. We show that mtDNA mutation levels inversely correlate with fertility, interestingly mainly affecting not male but female fertility. mtDNA mutations decrease female mice's fertility by reducing ovarian primordial and mature follicles. Mechanistically, accumulation of mtDNA mutations decreases fertility by impairing oocyte's NADH/NAD^+^ redox state, which could be rescued by nicotinamide mononucleotide treatment. For the first time, we answer the fundamental question of the causal effect of age‐accumulated mtDNA mutations on fertility and its sex dependence, and show its distinct metabolic controlling mechanism.

## Graphical abstract

Oocyte accumulates more mtDNA point mutations than sperm during aging, thus exacerbating female’s fertility through reducing its NADH/NAD+ redox ratio, which could be rescued by NMN.

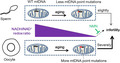

## 1 INTRODUCTION

Aging is one of the key factors in both male fertility and female fertility. Indeed, female fertility normally peaks at age 24 and diminishes after 30, with pregnancy occurring rarely after 50 (Goswami, & Conway, 2005). Alternatively, the rapid environmental changes also possibly contribute to the sharp increase in infertility and subfertility rates seen in recent years (Sharpe, & Franks, 2002). Mitochondrial malfunction has been hypothesized to play important roles in age‐ and environment‐induced infertility (Benkhalifa et al., 2014; Demain, Conway, & Newman, 2017). For instance, mitochondrial DNA (mtDNA) deletions were reported to accumulate in human ovarian aging (Gibson, Pei, Quebedeaux, & Brenner, 2006). Furthermore, mtDNA mutations may cause male infertility due to loss of spermatocytes and spermatids (Carra, Sangiorgi, Gattuccio, & Rinaldi, 2004; Kao, Chao, & Wei, 1995). As a result, assessment of mitochondrial function status (Santos, El Shourbagy, & St John, 2006; Sousa et al., 2011), mtDNA content, and mtDNA integrity (Babayev et al., 2016; Gibson et al., 2006; Tao et al., 2017) is often performed to investigate the quality of sperms and oocytes in assisted reproductive technologies. However, the links among aging, mtDNA mutations, and infertility remain not fully understood.

mtDNA mutations could result in mitochondrial dysfunction and thus metabolic disorders through compromising oxidative phosphorylation (OXPHOS). The nicotinamide adenine dinucleotide (NAD^+^)/reduced NAD^+^ (NADH) couples are known to play central roles in not only OXPHOS, but also other metabolic pathways such as glycolysis, the tricarboxylic acid cycle, and fatty acid oxidation. During oocyte maturation, activities of glycolysis and pentose phosphate pathway (PPP) were elevated within the oocyte cytoplasm (Cetica, Pintos, Dalvit, & Beconi, 2002; Downs, & Utecht, 1999; Tsutsumi, Satoh, Taketani, & Kato, 1992; Xie et al., 2016). These metabolic adaptations may be required for the production of biosynthetic precursors necessary for the rapid proliferation of an oocyte into an embryo following its fertilization. However, the role of NADH/NAD^+^ redox state in aging‐related infertility remains unclear.

mtDNA‐mutator (*PolgA^Mut^* ^/^ *^Mut^*) mice are widely used as an accredited experimental model to study the roles of mtDNA mutations in aging process. The *PolgA^Mut^* ^/^ *^Mut^* mice harbor a homozygous D257A mutation in the nuclear DNA‐encoded mitochondrial polymerase PolgA, leading to the inactivation of its proofreading function (Hauser, Primiani, Langston, Kumaran, & Cookson, 2015; Kim et al., 2019; Nissanka, Bacman, Plastini, & Moraes, 2018; Ross et al., 2013; Safdar et al., 2016; Vermulst et al., 2007, 2008). As compared to wild‐type (WT) mice, the *PolgA^Mut^* ^/^ *^Mut^* mice exhibited a ~10‐fold higher mtDNA mutation frequency, eventually leading to a progressive decline in the function of mtDNA‐encoded respiratory complexes. *PolgA^Mut^* ^/^ *^Mut^* mice were reported to show a reduced life span that is limited to 13–15 months (Chen et al., 2009; Norddahl et al., 2011). Consistently, aging‐associated disorders including cardiomyopathy, diabetes, reduced subcutaneous fat, alopecia, progressive hair graying, kyphosis (curvature of the spine), anemia, and osteoporosis occurred approximately 6–8 months after the birth of the *PolgA^Mut^* ^/^ *^Mut^* mice (Kujoth et al., 2005; Trifunovic et al., 2004). In the present study, we first determined how mtDNA mutations in human female oocytes changed with age. Using the *PolgA^Mut^* ^/^ *^Mut^* mouse model, we demonstrate mtDNA mutations decrease the fertility of females, but not males, via specific follicle defects. We further show that accumulation of mtDNA mutations decreases female fertility by reducing oocyte's NADH/NAD^+^ ratio and that nicotinamide mononucleotide (NMN) is remarkably capable of ameliorating infertility in female *PolgA^Mut^* ^/^ *^Mut^* mice.

## 2 RESULTS

### 2.1 Oocytes of female patients accumulate more mtDNA point mutations during aging

**FIGURE 1:**
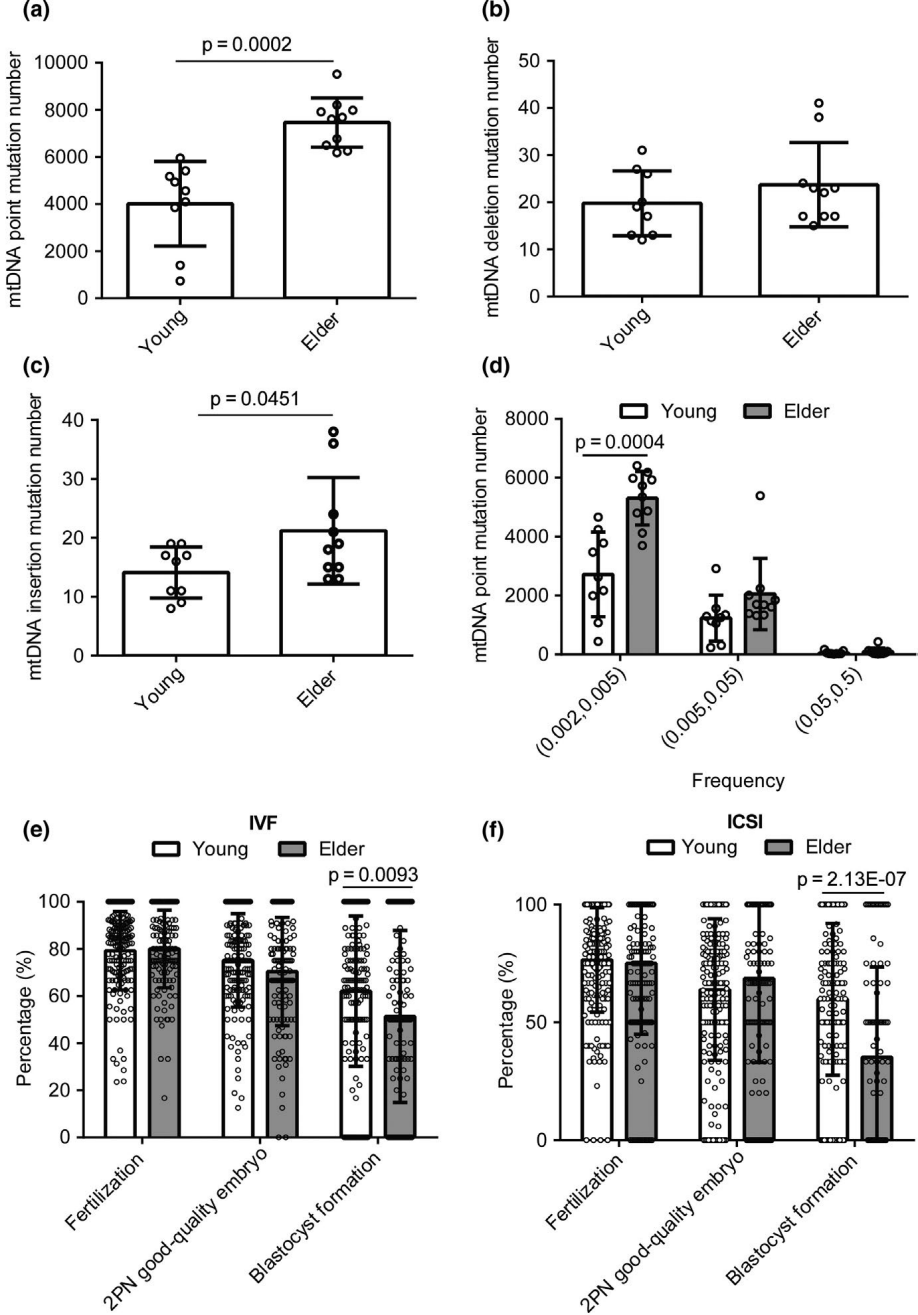
Elder female patients of age ≥38 years have lower blastocyst formation rate and more mtDNA point mutations in oocytes than young female patients of age ≤30 years. (a–d) Number of mtDNA point mutations with frequency >0.002 (a) mtDNA deletions with frequency >0.002 (b) mtDNA insertions (c) with frequency >0.002, and mtDNA point mutations with frequency with 0.05–0.5, 0.005–0.05, and 0.002–0.005 (d) among 16,569‐bp human mtDNA sequence (NC_012920.1) in discarded oocytes from young female patients (*n* = 9) and elder female patients (*n* = 10). 0.05–0.5 means frequency from 0.05 up to 0.5. (e) Fertilization rate (%) (*n* = 157 in young group and *n* = 133 in elder group), 2PN good‐quality embryo rate (%) (*n* = 157 in young group and *n* = 132 in elder group), and blastocyst formation rate (%) (*n* = 157 in young group and *n* = 133 in elder group) in young female patients (*n* = 157) and elder female patients (*n* = 133) with IVF; (f) fertilization rate (%) (*n* = 199 in young group and *n* = 190 in elder group), blastocyst formation rate (%) (*n* = 157 in young group and *n* = 103 in elder group), and 2PN good‐quality embryo rate (%) (*n* = 195 in young group and *n* = 171 in elder group) in young female patients (*n* = 199) and elder female patients (*n* = 190) with ICSI. Error bars are *SD*, and *p*‐values were calculated using unpaired two‐tailed Student's *t* test

In humans, female fertility begins to decrease after the age of 30 and decreases more rapidly after 37 (American College of Obstetricians & Gynecologists Committee on Gynecologic Practice & Practice Committee, 2014; van Noord‐Zaadstra et al., 1991). Based on this, we divided female patients undergoing in vitro fertilization (IVF) or intracytoplasmic sperm injection (ICSI) into young (≤30 years) and elder (≥38 years) groups, and investigated the impact of age on oocyte mtDNA mutations. Immature oocytes discarded during ICSI from female patients in the young (age = 27.4 ± 1.1, *n* = 9) and elder (age = 40.8 ± 0.8, *n* = 10) groups were analyzed for point, deletion, and insertion mutations in the mtDNA. Considering a false‐positive rate at 0.001 from sequencing system errors, the nucleotides with mutation frequency >0.002 were used for mtDNA mutation analysis. As shown in Figure 1a–c, discarded oocytes in the elder group (*n* = 10) had more mtDNA point and insertion mutations in the 16,569‐bp human mtDNA sequence (NC_012920.1) than found in the young group (*n* = 9). The number of mtDNA point mutations (more than 4000 in each group) was much greater than that of deletion and insertion mutations (less than 25 in each). We then sorted the mtDNA point mutations according to their frequency into three bins: 0.002–0.005, 0.005–0.05, and 0.05–0.5. We found that the elder group had much more, low‐frequency mtDNA point mutations (0.002–0.005) than the young group (Figure 1d).

Next, the fertilization rates, 2 pronuclear (2PN) good‐quality embryo rates and blastocyst formation rates, were determined for 157 cases from the young group (age = 27.8 ± 0.2) and 133 cases from the elder group (age = 39.8 ± 0.2) of patients undergoing IVF, and 199 cases of young group (age = 27.8 ± 0.1) and 190 cases of elder group (age = 41.3 ± 0.2) of patients with ICSI. Fertilization rates and 2PN good‐quality embryo rates showed no significant differences between young and elder, but blastocyst formation rate of elder group was significantly lower than that of young group in both IVF and ICSI cycles (Figure 1e,f). This finding is consistent with a previous study showing that blastocyst formation declined with age (Luna et al., 2009). Collectively, our results indicate that elder female patients have defects in blastocyst formation correlated with an increased accumulation of oocyte mtDNA point mutations.

### 2.2 Accumulation of mtDNA mutations in polgA mutator mice affects fertility in a sex‐ and age‐dependent manner

**FIGURE 2:**
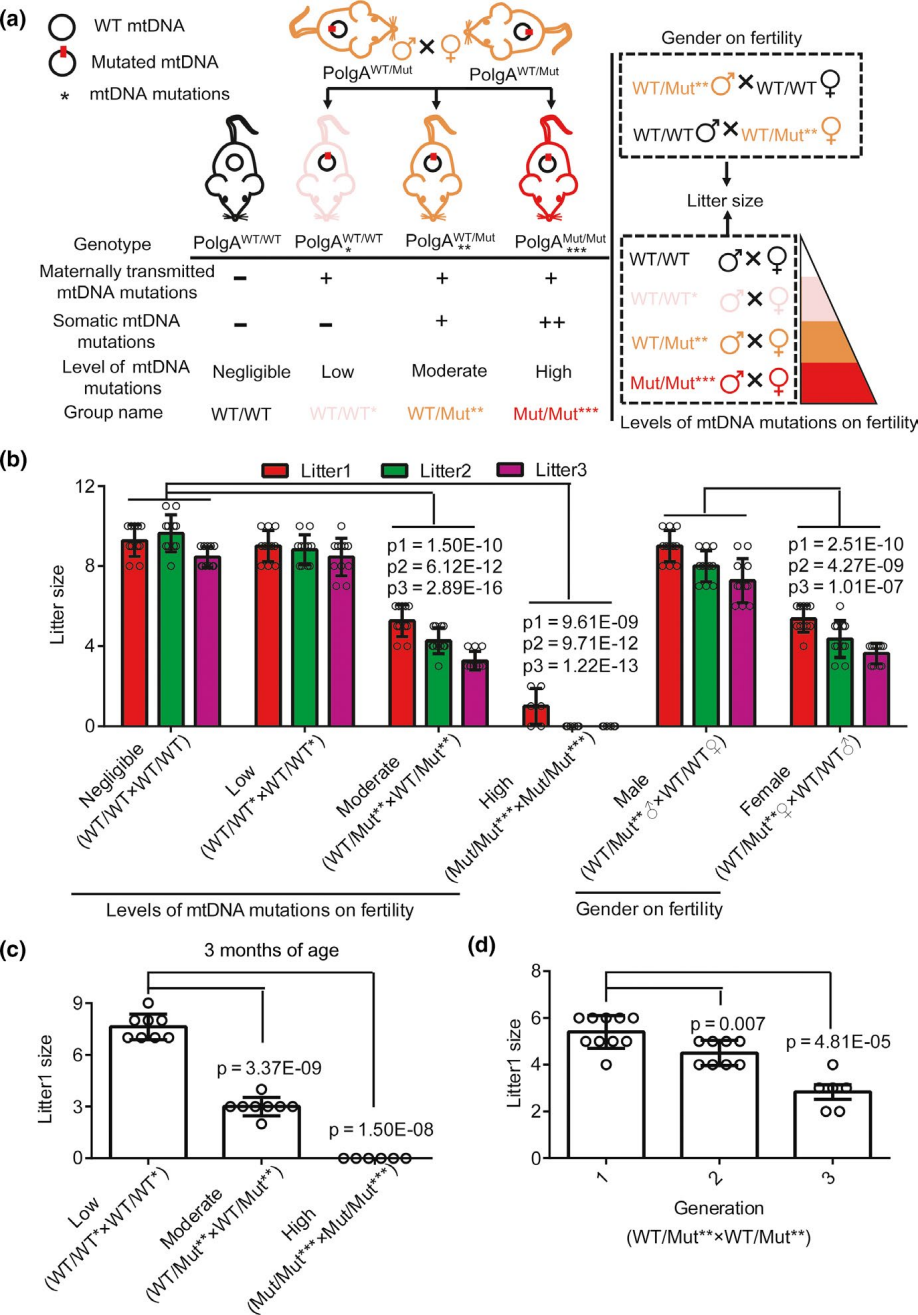
Accumulation of mtDNA mutations in PolgA mutator mice affects fertility in a sex‐ and age‐dependent manner. (a, b) The mean litter sizes of the first, second, and third litters per breeding pair at 7–8 weeks of age as indicated in the legends. The breeding pairs were generated as shown in a. The litter sizes were shown in b (*n* = 6 pair in Mut/Mut***×Mut/Mut*** breeding group, and *n* = 11 pair in the other breeding pairs). The values of *p*1, *p*2, and *p*3 were calculated using litters 1, 2, and 3 from WT/WT×WT/WT breeding pair as control. (c) The mean number of pups in the first litters per breeding pair at 3 months of age as indicated in the legends (*n* = 6 pair in Mut/Mut***×Mut/Mut*** breeding group, and *n* = 8 pair in the other breeding groups). (d) The mean number of pups in the first litters per WT/Mut**×WT/Mut** breeding pair at generations F1 to F3 as indicated in the legends (*n* = 10 pair in F1, *n* = 8 pair in F2 and *n* = 6 pair in F3). Error bars are SD, and *p*‐values were calculated using one‐way ANOVA test

To investigate the role of the level of mtDNA mutations in mammalian fertility, we generated a series of mice with increasing levels of mtDNA mutations, indicated by the number of asterisks (*), using *PolgA^WT^* ^/^ *^Mut^* and *PolgA^WT^* ^/^ *^WT^* mice (Figure 2a). *PolgA^WT^* ^/^ *^WT^* mice (WT/WT) showed negligible level of mtDNA mutations. *PolgA^WT^* ^/^ *^Mut^* mice were crossed to generate three types of offsprings with maternally transmitted mtDNA mutations, that is, PolgA^WT/WT*^, PolgA^WT/Mut**^, and PolgA^Mut/Mut***^ mice. Among these, PolgA^WT/WT*^ mice (WT/WT*) harbored only maternally transmitted mtDNA mutations. In addition to maternally transmitted mtDNA mutations, both PolgA^WT/Mut**^ (WT/Mut**) and PolgA^Mut/Mut***^ (Mut/Mut***) harbored somatic mtDNA mutations induced by *POLG* mutation. Thus, we generated mice of four levels of mtDNA mutations: negligible (WT/WT), low (WT/WT*), moderate (WT/Mut**), and high (Mut/Mut***). As shown in Figure 2b, compared to WT/WT×WT/WT breeding pairs, WT/WT*×WT/WT* breeding pairs with maternally transmitted mtDNA mutations showed no significant difference in litter size of the first, second, or third litter. WT/Mut**×WT/Mut** breeding pairs showed reduced fertility compared to WT/WT×WT/WT breeding pairs in all three litters. Further, Mut/Mut***×Mut/Mut*** breeding pairs showed little fertility compared to WT/WT×WT/WT breeding pairs. Moreover, in the second and third litters of Mut/Mut***×Mut/Mut*** breeding pairs, there were no offspring. (Figure 2b) Thus, our results demonstrate the quantitative correlation between mtDNA mutation level and fertility (Figure 2b).

Then, we asked which sex is primarily responsible for this reduced fertility. We crossed the male or female WT/Mut** to WT/WT mice. WT/Mut** male×WT/WT female breeding pairs showed no difference comparing to WT/WT×WT/WT breeding pair, whereas WT/Mut** female×WT/WT male breeding pairs showed significant decrease in litter sizes comparing to WT/WT×WT/WT breeding pairs (Figure 2b), indicating that moderate mtDNA mutations mainly affect fertility of female mice and not of male ones.

To further confirm our observations on mtDNA mutation accumulation and fertility, we crossed mice with different levels of mtDNA mutations, low (WT/WT*), moderate (WT/Mut**), and high (Mut/Mut***), at a later age‐3 months rather than 7–8 weeks. We found that the first litter size decreased in all three conditions, among which WT/Mut**×WT/Mut** breeding pair showed much more reduced fertility, and Mut/Mut***×Mut/Mut*** had no offspring at all (Figure 2c). Moreover, to determine the effects of mtDNA mutation accumulation across generations we recorded the first litter sizes of F1, F2, and F3 crosses of WT/Mut**×WT/Mut** breeding pairs at age 7–8 weeks. We found that fertility decreased in successive generations (Figure 2d). These results indicate that the accumulated mtDNA mutations occurring in PolgA mutator mice during aging and across generations decrease fertility.

### 2.3 Accumulation of mtDNA mutations in polgA mutator mice decreases ovarian primordial and mature follicles

**FIGURE 3:**
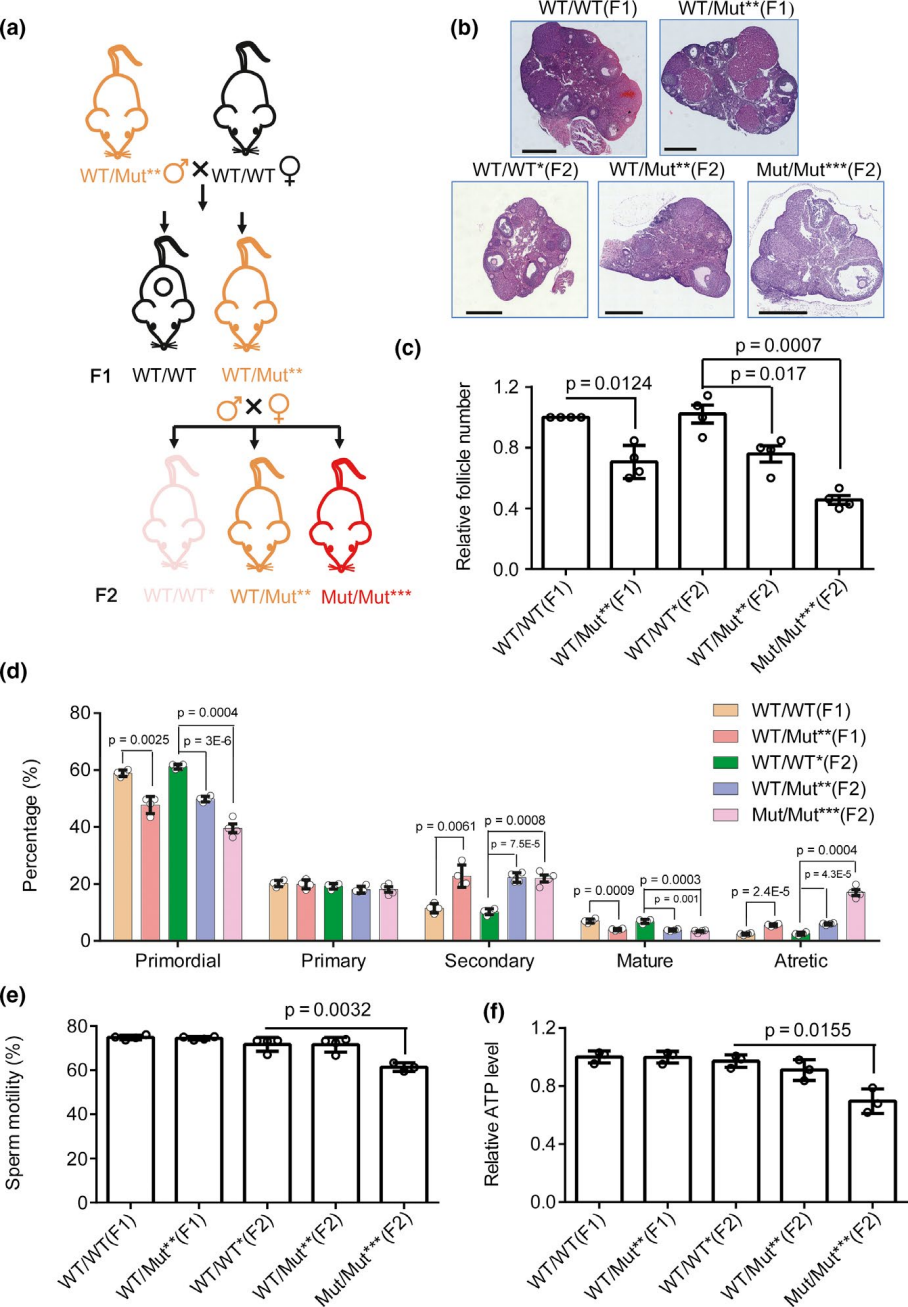
The effect of mtDNA mutations on follicles and sperm of mice. (a) Diagram for generating WT/WT(F1), WT/Mut**(F1), WT/WT*(F2), WT/Mut**(F2), and Mut/Mut***(F2) mice. (b–f) Ovary histopathology stained with H&E (b, Scar bar, 500 μm), relative ovarian follicle number (c), and classification of ovarian follicles (d) in female mice at 7–8 weeks of age as indicated in the legends. Sperm motility (e) and relative ATP level of sperm (f) in male mice at 7–8 weeks of age as indicated in the legends. *n* ≥ 4 for each group. Error bars are *SD*, and *p*‐values were calculated using one‐way ANOVA test

To further investigate how mtDNA mutations mainly affect female fertility, we firstly quantified the weights of reproductive organs. WT/Mut** males were crossed with WT/WT females to obtain WT/WT (F1) and WT/Mut** (F1) without maternally transmitted mtDNA mutations. WT/WT* (F2), WT/Mut** (F2), and Mut/Mut*** (F2) mice with maternally transmitted mtDNA mutations were then obtained by crossing WT/Mut** males (F1) to WT/Mut** females (F1) (Figure 3a). We weighed the body, testes, and epididymides of the males of these five genotypes at age 7–8 weeks and did not observe significant differences (Figure S1A,B). For female mice, we detected the number and classification of ovarian follicles in the ovaries. We found that the amount of ovarian follicles of WT/Mut** (F1) female mice was less than that of WT/WT (F1), and Mut/Mut*** (F2) female mice had fewer ovarian follicles compared to WT/WT* (F2) or WT/Mut** (F2) female mice (Figure 3b,c). These results indicate accumulated mtDNA mutations decrease ovarian follicles. Ovaries of WT/Mut** (F1) mice had fewer primordial and mature follicles, and more secondary follicles and antral follicles than that of WT/WT (F1). Ovaries of Mut/Mut*** (F2) or WT/Mut** (F2) mice had less primordial and mature follicles, and more secondary follicles and antral follicles than that of WT/WT* (F2) mice (Figure 3d). These results indicate accumulated mtDNA mutations cause aging of ovary. As shown in Figure S1C, the ratio of growing follicles (i.e., primary and secondary follicles) to primordial follicles in Mut/Mut*** (F2) was significantly higher than that in WT/WT* (F2), suggesting an over‐activation of the ovarian follicle in Mut/Mut*** (F2). This could lead to follicle depletion and ovarian “burn‐out,” and eventually to infertility (Kalich‐Philosoph et al., 2013).

For male mice, we performed testes histopathology stained with H&E, and evaluated number, aberration rates, motility, and relative ATP levels of sperm. We did not observe differences in testes histopathology (Figure S1D), number, and aberration rate of sperm (Figure S1E,F) among these five groups. However, sperm of Mut/Mut*** (F2) male mice with most mtDNA mutations than other groups have lower mobility and ATP levels than sperm of WT/WT* (F2) male mice, while WT/Mut** (F1/F2) male mice do not show difference in mobility and ATP level of sperm comparing to WT/WT* (F1/F2) (Figure 3e,f). These results indicate that only robust mtDNA mutation rates can cause decreased mobility and ATP levels of sperm.

### 2.4 Accumulation of mtDNA mutations decreases oocyte's NADH/NAD^+^ redox state

**FIGURE 4:**
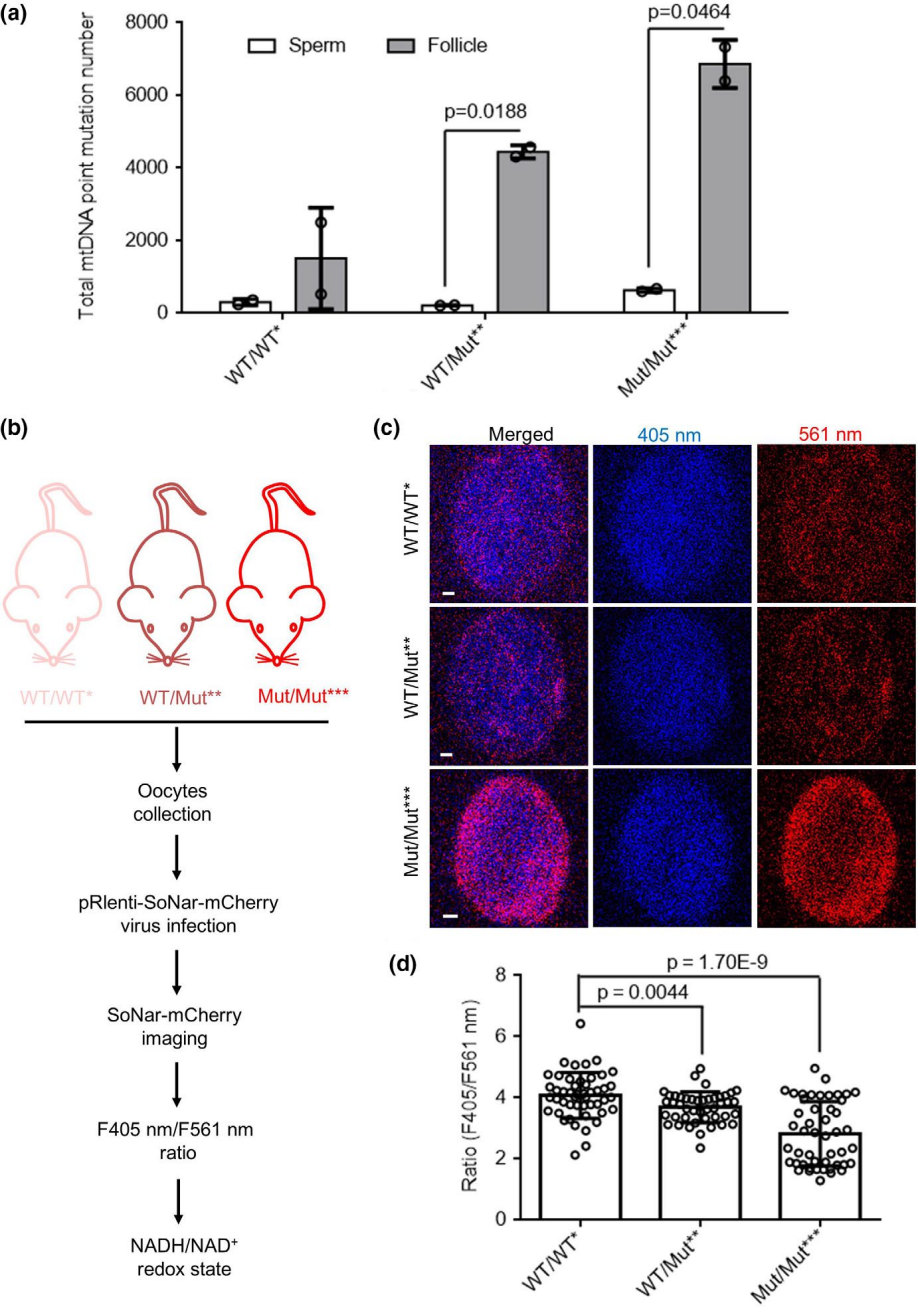
Accumulation of mtDNA mutations decreases oocyte's NADH/NAD^+^ redox state. (a) The total point mutations with frequency >0.002 in sperm and follicle (*n* = 2 mice for each genotype) in WT/WT*, WT/Mut**, and Mut/Mut*** mice at age 7–8 weeks. (b) Diagram for detecting NADH/NAD^+^ redox state of oocytes from WT/WT*, WT/Mut**, and Mut/Mut*** mice. (c) The representative images of the ratios of SoNar fluorescence of oocytes from WT/WT*, WT/Mut**, and Mut/Mut*** mice (Scale bar, 5 μm). (d) Quantification of the fluorescence ratios in C (*n* ≥ 44 oocytes collected from two female mice for each genotype). Error bars are *SD*, and *p*‐values were calculated using one‐way ANOVA test

Results in Figures 2, 3 showed that there is no significant difference in fertility, sperm activity, or follicle number between WT/WT (F1)/WT/Mut** (F1) and WT/WT* (F2)/WT/Mut** (F2). The mtDNA mutations in sperm and follicle of WT/WT*, WT/Mut**, and Mut/Mut*** mice obtained by crossing WT/Mut** males to WT/Mut** females were further analyzed to investigate the types of mtDNA mutations: deletion, insertion, and point mutations. First, we compared mtDNA mutations of the follicle and sperm in these three groups at age 7–8 weeks. We showed that follicles in WT/Mut** and Mut/Mut*** had more point mutation sites among the whole mtDNA genome comparing to sperm (Figure 4a). Follicles in WT/WT* mice had fewer deletion mutation sites than sperm, while follicles in WT/Mut** mice had fewer deletion and insertion mutation sites (Figure S2A,B). However, the number of deletion and insertion mutation sites in follicles of these three groups was much less than that of point mutation sites, which is consistent with our findings in human oocytes.

We further compared the frequency of mtDNA point mutations in follicles and sperm in these three groups. We found that follicles of Mut/Mut*** mice had more mtDNA point mutations of frequency among (0.005–0.05) than WT/WT* mice, while sperm of Mut/Mut*** mice had more mtDNA point mutations with frequency among (0.002–0.005) than WT/WT* mice. The above results showed that follicles accumulate more mtDNA point mutations than sperm (Figure S2C,D).

**FIGURE 5:**
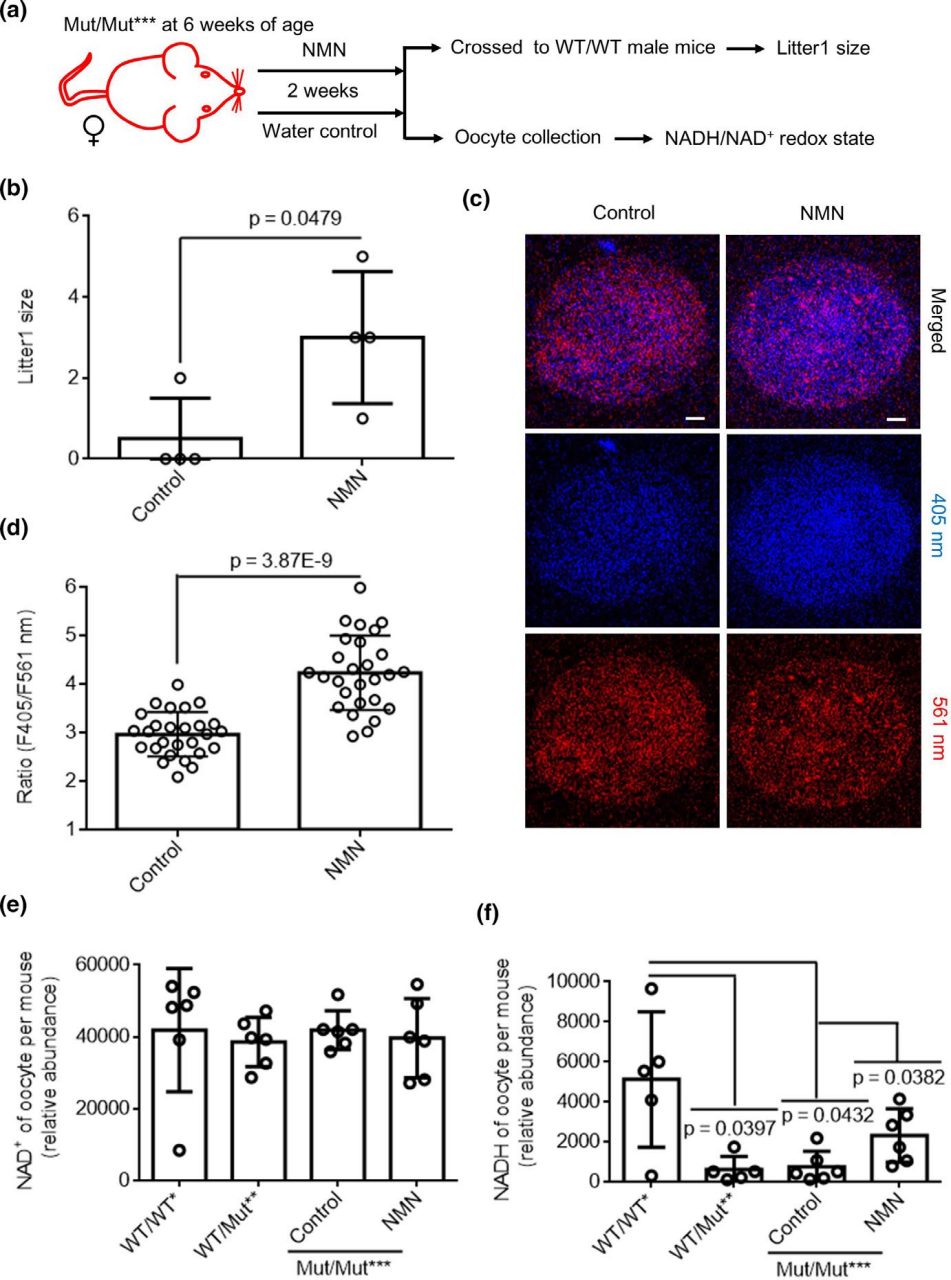
Accumulation of mtDNA mutations decrease female fertility by reducing oocyte's NADH/NAD^+^ redox state. (a) Diagram for detecting NADH/NAD^+^ redox state of oocytes from Mut/Mut*** female mice crossed to WT/WT male mice with NMN or water. (b–d) The first and second litter size (b, *n* = 4 breeding pair for each genotype), representative images of the ratios of SoNar fluorescence of oocytes (c, *n* ≥ 27 oocytes collected from two female mice for each group, Scale bar, 5 μm) and their quantification of the fluorescence ratios (d) of Mut/Mut*** female mice crossed to WT/WT male mice with NMN or water. Measurement of NAD^+^ (e) and NADH (f) levels in 10 oocytes per mouse in WT/WT* (*n* = 6 for NAD^+^, and *n* = 5 for NADH), WT/Mut** (*n* = 6 for NAD^+^, and *n* = 5 for NADH), and Mut/Mut*** treated with NMN (*n* = 6 for NAD^+^, and *n* = 6 for NADH) or water (*n* = 6 for NAD^+^, and *n* = 6 for NADH) using Mass spectrometry. WT/WT* mice were used as control, and *n* = 6 mice for each group. Error bars are *SD*, *p*‐values were calculated using one‐way ANOVA test for e and f, and using unpaired two‐tailed Student's *t* test for b and d

NADH/NAD^+^ has an important role in energy metabolism, and their redox state can be monitored in living cells using SoNar, a NADH/NAD^+^ sensor. (Zhao et al., 2015, 2016) To study the effect of mtDNA mutations on NADH/NAD^+^ redox state in oocytes, we ectopically expressed SoNar in oocytes from WT/WT*, WT/Mut**, and Mut/Mut*** mice, and determined the ratios of fluorescence intensity (FI) excited at 405 and 561 nm (Figure 4b). The results showed that the F405/561 ratios of SoNar in oocytes of Mut/Mut*** mice are lower than that of WT/WT* mice. (Figure 4c,d) We next employed mass spectrometry to quantify the levels of NADH and NAD^+^, respectively. The results revealed a dramatic decrease in the amount of NADH in both WT/Mut** and Mut/Mut*** as compared to WT/WT* mice (Figure 5e,f). NADH serves as a vital redox‐energy currency for energy production in mitochondria. As expected, oocytes of Mut/Mut*** mice exhibited lower ATP levels than WT/WT mice (Figure S2E). These data indicate that oocytes of Mut/Mut*** mice have lower NADH/NAD^+^ redox ratio and weaker energy production.

As female fertility is affected by aging in addition to the above‐mentioned mtDNA mutations, we further detected oocyte's NADH/NAD^+^ redox ratio in young and elder mice. We found a significantly decreased NADH/NAD^+^ ratio in elder mice as compared to the young counterparts (Figure S2F).These results thus established a link between female infertility and perturbed NADH/NAD^+^ redox state.

### 2.5 Accumulation of mtDNA mutations decreases female fertility by impairing oocyte's NADH/NAD^+^ redox state

Whether the low fertility of Mut/Mut*** mice is due to lower NADH/NAD^+^ redox state needs to be investigated. As it has been reported that NMN is a promising therapy for aging‐associated physiological dysfunction and diseases through rescuing NADH/NAD^+^ redox state (Caton, Kieswich, Yaqoob, Holness, & Sugden, 2011; Imai, 2010), we tested whether NMN could increase fertility of mice with high levels of mtDNA mutations. We treated Mut/Mut*** mice with NMN for 2 weeks. After the Mut/Mut*** female mice were crossed with WT/WT male mice, the size of the first litter was evaluated (Figure 5a). As shown in Figure 5b, the first litter size of Mut/Mut*** female treated with NMN was higher than that from Mut/Mut*** female with water. This result indicates that NMN is remarkably capable of ameliorating infertility in Mut/Mut*** female mice. We further measured F405/561 ratios of SoNar in oocytes of Mut/Mut*** female mice treated with NMN or water (Figure 5a), and observed that the F405/561 SoNar ratios in oocytes of Mut/Mut*** mice with NMN were higher than that with water (Figure 5c,d), demonstrating an enhancement of the NADH/NAD^+^ ratio by NMN treatment. Respective quantification of NADH and NAD^+^ revealed an increase in the amount of NADH, but not of NAD^+^, in oocytes of Mut/Mut*** treated with NMN (Figure 5e,f). We also noted that mtDNA point mutations of the follicle of Mut/Mut*** female mice were unaffected by NMN treatment (Figure S3A). Given that NMN could also enhance oocytes' quality (Bertoldo et al., 2020), we interrogated whether the enhancement of fertility by NMN treatment could be due to differences in ovarian development. Interestingly, NMN treatment did not seem to alter the number of primordial and mature follicles in Mut/Mut*** female mice (Figure S3B). NMN treatment was shown to induce mitophagy in stem cells, leading to removal of dysfunctional mitochondria and thus cell function recovery (Fang et al., 2014; Lautrup, Sinclair, Mattson, & Fang, 2019; Vannini et al., 2019), we further investigated whether the effect of NMN on fertility could result from mitophagy induction. To test this possibility, we performed immunofluorescence of microtubule‐associated protein 1 light chain 3 (LC3) (Kabeya et al., 2000; Klionsky, Cuervo, & Seglen, 2007) to mark autophagosomes and used Mito Tracker Deep Red to stain mitochondria in oocytes (Figure S3C). The results showed that NMN treatment did not seem to change the number of mitophagosomes in oocytes (Figure S3D).

However, the possible dissipation of mitochondrial membrane potential during mitophagy could promise the use of this dye to quantify mitophagosomes. Interestingly, NMN failed to alter the motility and ATP levels of sperm in Mut/Mut*** male (Figure S3E,F). All these results indicate that NMN can rescue fertility of PolgA mutator mice with more point mutations by enhancing cellular NADH/NAD^+^ ratio in oocytes.

## 3 DISCUSSION

For the first time, we quantified the effect of aging on the accumulation of heteroplasmic mtDNA mutations in human individual oocytes using next‐generation sequencing (NGS). Aging of the human female reproductive system is much faster than that of other body systems, and follicle number reduction and oocyte quality decay with oxidative damage during ovarian aging cause the gradual decline in female fertility (Baird et al., 2005; Wang, Qi, Qi, Tang, & Shen, 2020; Wang, Zheng, et al., 2020). For the hypothesis of mtDNA mutations in oocyte quality declining with ovarian aging (Barritt, Brenner, Cohen, & Matt, 1999), our work showed the mtDNA mutation types in oocytes during age. By deep sequencing, oocytes have been reported to be not prone to accumulate mtDNA heteroplasmic mutations during ovarian aging (Boucret et al., 2017). However, high‐throughput sequencing technology might miss the low‐frequency mutations lower than 2%, which were detected as most abundant mutations in elder female patients in the present paper. mtDNA copy number inversely correlates with implantation potential of euploid embryos (Fragouli et al., 2017; Ravichandran et al., 2017) and was used as a potential biomarker for embryo viability in assisted reproduction. Our study also showed that mtDNA point mutations inversely correlate with oocyte quality, which provides another potential biomarker for embryo viability in assisted reproduction. Indeed, mitochondrial transfer has been used to exchange and enhance the integrity, activity, and number of mitochondria in quality‐compromised oocytes, which was recently used to improve fertility in women with previous poor reproductive performance by autologous mitochondrial injection treatment (Woods, & Tilly, 2015). Our work provides a biomarker for the clinical application.

mtDNAs are generally believed to be exclusively maternally inherited in most animals, due to the breakdown of paternal mitochondria by autophagy upon fertilization (Zhou et al., 2016), which is a highly conserved eukaryotic cellular recycling process undergoes extensive post‐translational modifications (Wang, Qi, et al., 2020; Wang, Zheng, et al., 2020). In human, mtDNA is transmitted to offspring mostly through the maternal lineage (Wallace, 2007), a clinically asymptomatic female patients with low levels of deleterious mtDNA mutations, resulting in the degrade of oocyte quality. By using the POLG^D257A^ mouse model, we demonstrated that the accumulated mtDNA mutations also degrade oocyte quality by follicle number reductions but have little effect on sperm activity. Tissues of WT mice during aging accumulate significant mtDNA deletions, but tissues of POLG^D257A^ mice accumulate a 7‐ to 11‐fold higher level of mtDNA deletions (Vermulst et al., 2007), and a 2500‐fold higher level of point mutations compared to WT mice (Vermulst et al., 2008). The *POLG^D257A^* mice have been used as a model to address the role of mtDNA mutations in age‐related disorders of multiple tissues including hair, skeleton, blood, and heart (Trifunovic et al., 2004). This mouse model allows detailed examination of the causal role of mtDNA mutations in age‐related fertility and its dependence on sex. It has been reported that new mtDNA point mutations are made by replication errors during oogenesis (Otten et al., 2016), and the accumulation of mtDNA mutations affects oocyte quality in terms of the risk of transmitting mitochondrial abnormalities to the offspring (Sallevelt et al., 2017). mtDNA mutations and copy number reduction have been reported to be associated with oocyte aging (Babayev et al., 2016; Gibson et al., 2006; Tao et al., 2017). Our results answer the fundamental question—which step of oogenesis is damaged by age‐related mtDNA mutations—and suggested follicles could be the potential therapy target for female infertility. For sperm aging, mtDNA mutations may cause male infertility due to loss of spermatocytes and spermatids which can be rescued by increasing total mtDNA copy number (Jiang et al., 2017). However, our results demonstrate that mtDNA mutations specially reduce sperm motility without significantly compromising the fertility of young male. The levels of mtDNA mutations in oocytes versus other cell types, and the levels of heteroplasmy in offspring are a worthy follow‐up study, thus being a limitation of our present study.

NAD^+^/NADH redox state is known to play essential roles in cell metabolism. The expression of SoNar (Zhao et al., 2015) has been widely used to measure the metabolic state of a single living cell for its intense fluorescence, rapid response, and pH‐resistant property (Oldham, Clish, Yang, & Loscalzo, 2015; Sullivan et al., 2015; Titov et al., 2016). Using this methodology, we demonstrated that mtDNA mutations decrease female POLG mutator mice's fertility by impairing oocyte's NADH/NAD^+^ redox state. Mass spectrometry further revealed a dramatic decrease in the amount of NADH in oocytes of POLG mutator mice. We reason that the high levels of mtDNA mutations may compromise mitochondrial respiration through mechanisms such as mtDNA methylation (Dou et al., 2019), eventually leading to the perturbed NADH/NAD^+^ redox state. Notably, the oocytes from elder mice also showed a similarly perturbed NADH/NAD^+^ redox state. Interestingly, NAD^+^ availability was recently shown to decrease with age (Bertoldo et al., 2020). Hence, our study further emphasizes important roles of NADH/NAD^+^ redox state in oocyte aging. The perturbed NADH/NAD^+^ redox state may further compromise energy metabolism, as is observed for the oocytes of POLG mutator mice.

NMN, a key NAD^+^ intermediate, has been shown to enhance NAD^+^ biosynthesis, activate SIRT1, and improve metabolic and stress responses in aging mice as well as ameliorate various pathologies in mouse disease models (Mills et al., 2016; Yoshino, Mills, Yoon, & Imai, 2011). Consistently, NMN treatment elevated the amount of NADH, the reduced form of NAD^+^, in oocytes of POLG mutator mice. The unchanged amount of NAD^+^ with the up‐regulation of NADH indicated that the overall size of NADH/NAD^+^ pool in oocytes of Mut/Mut*** was increased by NMN treatment. Thus, NMN can be considered as a potential agent in the treatment of cell metabolism disorders triggered by perturbed the overall of NADH/NAD^+^ pools. NMN supplementation has been reported to reverse age‐related arterial, vascular, and skeletal muscle dysfunction in mice by mitochondrial‐related signaling (de Picciotto et al., 2016; Mills et al., 2016). Indeed, it was shown to be transported into mammalian mitochondria (Davila et al., 2018). The short‐term administration of NMN has been reported to have remarkable therapeutic effects on metabolic complications and other disease conditions (Caton et al., 2011; de Picciotto et al., 2016). In short, NMN is viewed as a promising therapy for age‐associated physiological dysfunction and disease (Caton et al., 2011; Imai, 2010). We found NMN also has potential as a drug for mtDNA mutation caused oocyte aging. Further mechanistic studies are required in future.

**FIGURE 6:**
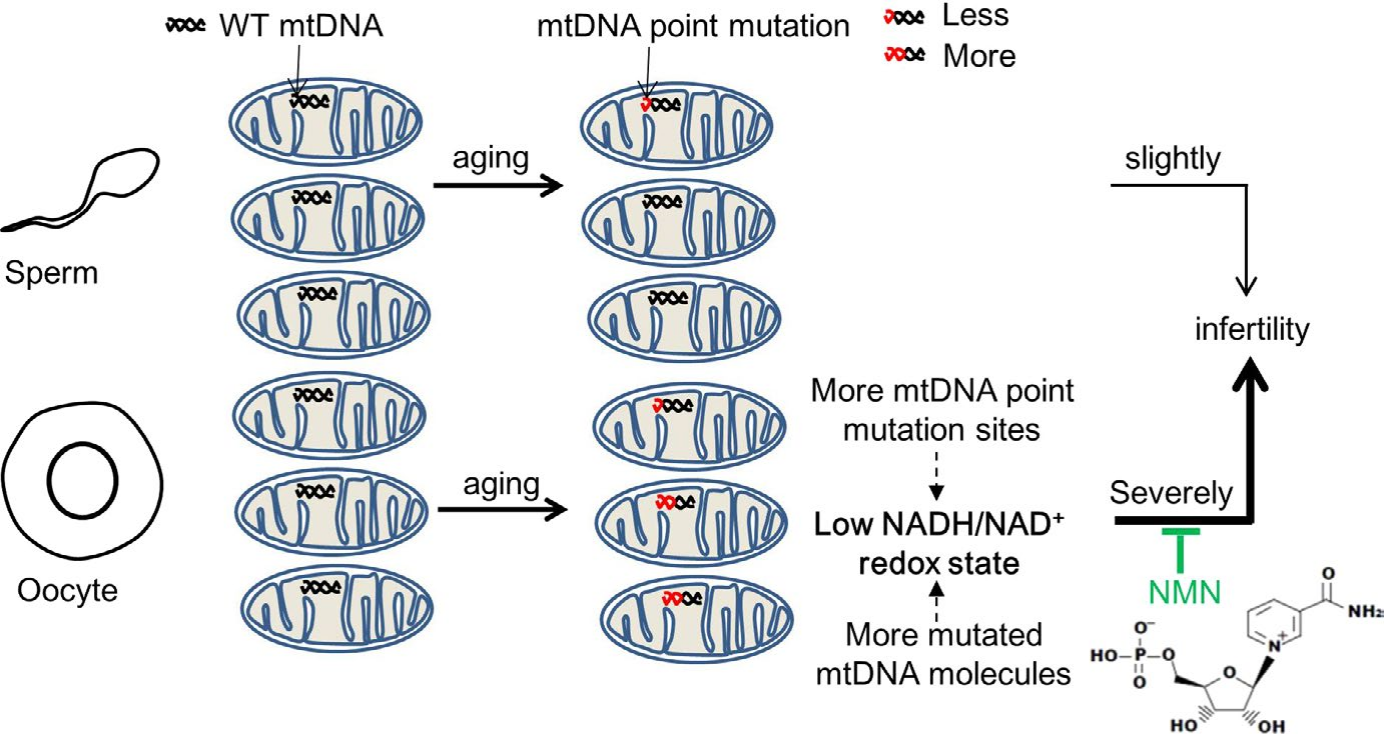
Model of the role of mtDNA point mutations in follicles and sperm on fertility during aging

In summary, our study, by systematically comparing the quality and mtDNA mutations of oocytes in young and elder female patients, showed that mtDNA point mutations inversely correlate with oocyte quality, which provides another potential biomarker for embryo viability in assisted reproduction, and demonstrated NMN as a potential candidate drug for oocyte aging caused by mtDNA mutation (Figure 6).

## 4 EXPERIMENTAL PROCEDURES

### 4.1 Case subject

Female patients with IVF/ICSI of The Sixth Affiliated Hospital of Sun Yat‐sen University (Guangzhou, China) were classified into young and elder groups. The general condition and clinical outcomes of 199 cases of young group and 190 cases of elder group patients undergoing ICSI from May 2017 to March 2018 and 157 cases of young group and 133 cases of elder group of patients undergoing IVF from December 2016 to January 2018 were analyzed, including ovary fertilization rate, blastocyst formation rate, and 2PN good‐quality embryo rate. These were approved by the Ethics Committee of The Sixth Affiliated Hospital of Sun Yat‐sen University (2017ZSLYEC‐016S).

### 4.2 Mice

*PolgA^D257A^* heterozygous male mice (*PolgA^WT^* ^/^ *^Mut^*) were purchased from the Jackson Laboratory (Stock No: 017341). The genotypes of siblings were determined by sequencing the genome of mice tail at 4 weeks old. All animal procedures followed Health guidelines. All experiments were performed in accordance with relevant guidelines and regulations, which had been reviewed and approved by the Guangzhou Institutes of Biomedicine and Health Ethical Committee (Approve no. 2013021).

### 4.3 Histology

Before histological studies, testis and ovaries were randomly excised from either the left or right side of each mouse in a weighed group. Testis and ovaries were collected and fixed in 4% paraformaldehyde for 24 hr, embedded in paraffin, and serially sectioned at a thickness of 4 μm. Paraffin sections of testis and ovary were mounted on slides and stained with hematoxylin–eosin (H&E) and observed under a light microscope (Zeiss).

### 4.4 Sperm preparation, counts, and motility

The epididymis was cut into small pieces and cultured to allow the sperm to swim up as described (Li et al., 2010; Sarkar, Chowdhury, & Singh, 2016). The number of spermatozoa was counted according to World Health Organization (1999) laboratory manual. Sperm motility was recorded within 7 min from dissection, and sperm motility parameters were subsequently recorded 2 min after loading the slides. Sperm abnormalities were further evaluated according to the criteria (Wyrobek, & Bruce, 1975) after sperm morphology was observed under a phase contrast microscope.

### 4.5 ATP detection

The amount of ATP in equal number of sperms or oocytes per mouse was detected using the ENLITEN ATP Assay System (Promega Corp.) according to the manufacturer's protocol.

### 4.6 Analysis of mtDNA mutations

For the experiment design, we considered the sample size of female patients for collecting discarded oocytes. Based on previous knowledge, we preset the standard deviation of outcome index to be 1500. After specifying *α* = 0.05 and *β* = 0.20 (for 80% power), we estimated that a total sample size of nine female patients for each group would be required. Based on the biological principles of oocyte development, only mature metaphase II (MII) oocytes were used in the ICSI procedure, whereas the immature metaphase I (MI) oocytes and germinal vesicle (GV) oocytes are usually discarded in ICSI cycles if the majority of the retrieved oocytes are mature. Following obtainment of written informed consent from the patient, 1–4 immature discarded oocytes during ICSI were obtained and a clinical evaluation was performed following protocols approved by the Ethics Committee of The Sixth Affiliated Hospital of Sun Yat‐sen University (2017ZSLYEC‐015S). For human oocyte mtDNA analysis, 1–4 immature oocytes discarded during ICSI from single female patient were collected as one sample, lysed in 20 μl lysis buffer (17.5 μl H_2_O, 2 μl 10× KOD buffer [TOYOBO], 0.5 μl proteinase K [TianGen]) at 56°C for 45 min, and then incubated at 95°C for 10 min to quench the enzyme activity of proteinase K. For human oocyte mtDNA analysis, the mixture was directly used as template of following PCR. Four pairs of primers were used to amplify region 3561–9794, region 9795–14,567, region 14,562–139, and region 115–3560, which cover the whole mtDNA sequence. The corresponding primers were listed in Table S1. Five microlitre template of oocytes from single patients was used to amplify each region of mtDNA using KOD Plus enzyme (TOYOBO). PCR of mtDNA was performed, and then, the four amplified sequences were mixed and sent for sequencing.

For mice mtDNA analysis, total genome of sperm or follicle isolated from mice ovary was prepared using a genome extraction kit (TianGen). Three pairs of primers were used to amplify region 1872–6222, 6203–10,627, and 10,622–1871, which cover the whole mtDNA sequence. The corresponding primers were listed in Table S2. Then, the three amplified sequences were mixed and sent for sequencing.

The resulting ddRAD (double‐digest restriction‐associated DNA) library was sent to Berry Genomics. Co., Ltd and sequenced on the Illumina Hiseq4000 platform using 150‐bp paired‐end reads for 3G flux. By using default parameters, the quality‐filtered reads of mtDNA from mice sperm and follicle were aligned to the reference mitochondrial genome of Mus musculus strain C57BL/6J mitochondrion (GenBank: AY172335.1), and the quality‐filtered reads of mtDNA from human oocytes were aligned to the reference *Homo sapiens* mitochondrion (GenBank: KC417443.1). Briefly, any putative point mutation site must meet the following criteria: (a) The nucleotide supporting the mutation should have a sequencing and mapping score greater than 30; (b) has a mutation frequency greater than 0.2%; and (c) the mutation site should also be supported by three or more best‐unique reads (BURs) and mutation frequency supported by best‐unique reads should be >0.2% to avoid sporadic sequencing errors due to higher coverage. The cutoff stringency of ≥3 BURs is adjusted based on average BUR coverage depth).

### 4.7 Ovarian follicle categorization and counting

The relative total number of follicles per ovary was determined by taking the average of the counts from three sections (five sections apart) cut along the long axis of the whole ovary. Follicle classification was determined by Pederson's system (Pedersen, 1970). The results were reported as the number of follicles counted per ovary.

### 4.8 Plasmid constructs

The SoNar‐mCherry plasmids were gifts from Professor Yi Yang and Yuzheng Zhao (East China University of Science and Technology, China). SoNar‐mCherry plasmid was then sub‐cloned into a lentiviral expression vector pRlenti (Wu et al., 2016).

### 4.9 Fluorescence microscopy

Oocytes from female mice at age of 7–8 weeks were collected using standard protocols as described (Seli et al., 2005). For SoNar imaging, oocytes were infected with pRlenti‐SoNar‐mCherry virus for 24–48 hr and then seeded on a 20‐mm glass‐bottom cell culture dish for image. The oocytes were imaged using a Zeiss LSM 710 with a 20x objective, and the ratios of F405 nm/F561 nm were obtained as described previously (Zhao et al., 2015, 2016). For detecting mitophagy, oocytes were incubated with MitoTracker Deep Red FM (Invitrogen, M22426, 1:5000) for 30 min and then performed immunofluorescence using anti‐LC3B antibody (Cell Signal Technology, 2775, 1:200) and Alexa Fluor 488‐conjugated secondary antibody (Life Technologies, A‐11,008, 1:400). Then, oocytes were imaged using a Zeiss LSM 710 with a 40x objective.

### 4.10 Measurement of NAD^+^ and NADH levels

Ten oocytes per mouse were harvested as described (Bustamante et al., 2017) and sent to Tsinghua University (Beijing, China) for determining NAD^+^ and NADH contents using a liquid chromatography‐tandem mass spectrometry (LC‐MS/MS).

### 4.11 NMN administration

Water consumption was measured for 2 weeks prior to the start of NMN (1094‐61‐7; Sigma) administration. NMN was then administered in drinking water at 900 mg/kg/day, based on the previously measured water consumption (Mills et al., 2016). The administration began at 6 weeks of age and continued for 2 weeks. The NMN solution was prepared weekly in small batches by dissolving NMN into autoclaved water at the certain dose and filtering sterilely. Water bottles and cages were changed twice weekly.

### 4.12 Statistical analysis

The data are shown as mean ± standard deviation (*SD*). All statistical analysis was performed using SPSS software (SPSS Inc., Chicago, IL, USA). Comparison in different genotype mice was made by one‐way ANOVA test as indicated in the legends. The significance of statistical differences between two groups was evaluated using the unpaired two‐tailed Student's *t* test as indicated in the legends. *p*‐values of less than 0.05 were considered as significant, and the exact *p*‐values were shown in the figures.

## CONFLICT OF INTEREST

The authors declare that they have no conflict of interest.

## AUTHOR CONTRIBUTIONS

X. Liu initiated and supervised the project. L.Y. and X. Lin performed the experiments and analyzed the data. H.T., Y.F., and Z.H. performed the experiments. L.J., Y.S., S.H., and X. Liang helped the human materials. Y.Y. provided suggestions. X. Liu, L.Y., and X. Lin wrote the manuscript.

## Supporting information

Supplementary Material

## Data Availability

The data that support the findings of this study are available from the corresponding author (X.L.), upon reasonable request.
