# Supplementary material for: Mitochondrial DNA mutation exacerbates female reproductive aging via impairment of the NADH/NAD+ redox
Source: Aging Cell. 2020 Aug 3;19(9):e13206. doi: 10.1111/acel.13206 (PMC7511885; doi:10.1111/acel.13206)
Supplement: Supplementary file 1 — Supplementary Material [file ACEL-19-e13206-s001.docx]

**
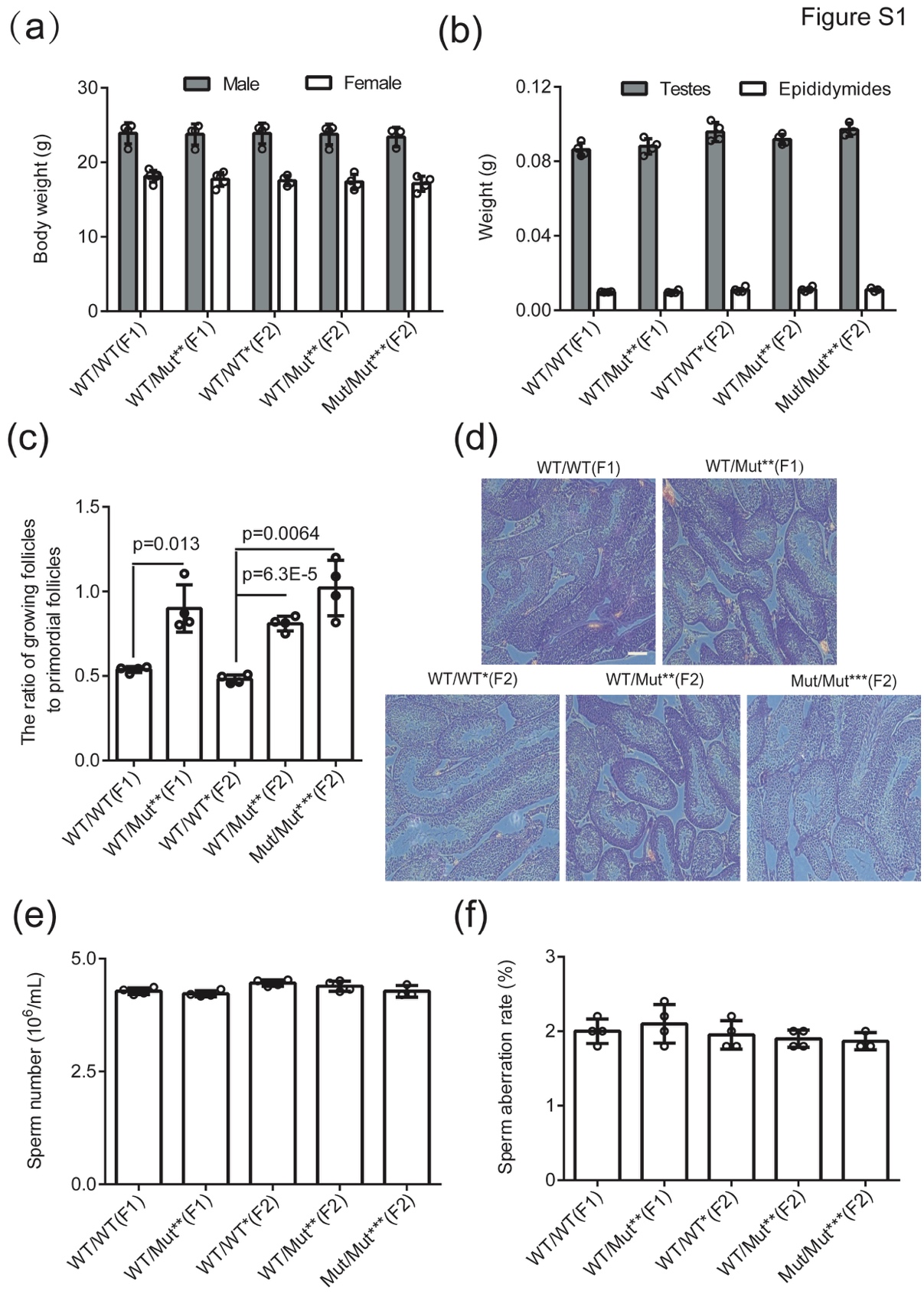
**

**Supplementary Figure 1, Related to Figure 3.** Body weight of male and female mice at age of 7-8 weeks (a), testes/epididymides weight (b), the ratio of growing follicles (primary and secondary follicles) to primordial follicles (c), testes histopathology stained with H&E (d, Scar bar, 100 μm), sperm number (e) and sperm aberration rate (f) of male mice at age of 7-8 weeks as indicated in the legends (n≥4 for each group). Error bars are SD. Error bars are SD, and P-values were calculated using one-way ANOVA test.

**
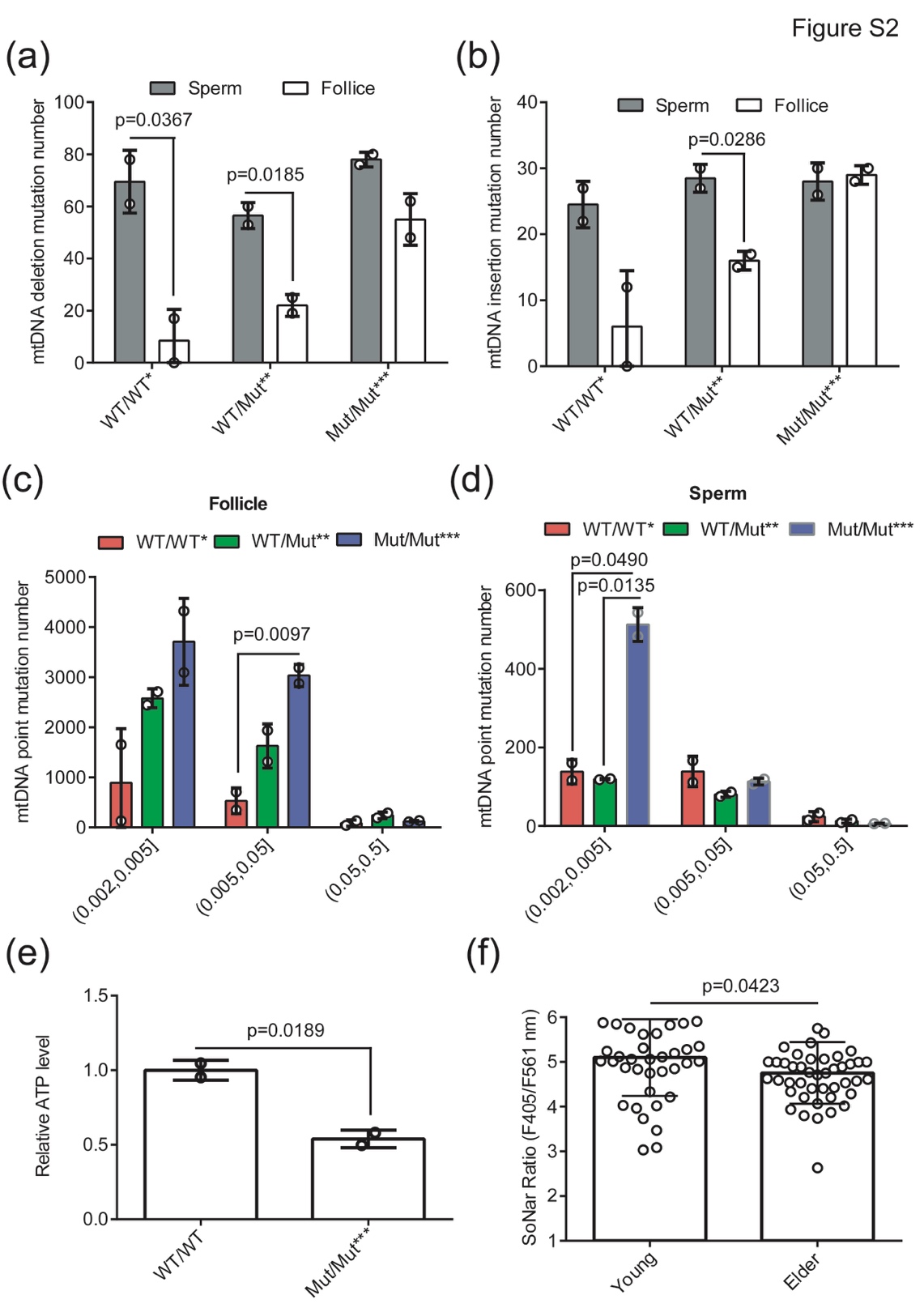
**

**Supplementary Figure 2, Related to Figure 4**. mtDNA deletion mutation number (a) and insertion mutation number (b) in follicle and sperm, mtDNA point mutations with (0.002-0.005), (0.005-0.05) and (0.05-0.5) frequency in follicle (c) and sperm (d) derived from Fig. 4A (n=2 for each gene-type). (e) Relative ATP levels in oocytes from WT/WT and Mut/Mut*** mice at age of 7-8 weeks (n=2). (f) Quantification of SoNar ratio (F 406/F 561) in oocytes from young WT mice at age 5-8 weeks (n=42 oocytes collected from 4 WT female mice) and elder WT mice at age 20-25 weeks (n=46 oocytes collected from 6 WT female mice). Error bars are SD, P-values were calculated using one-way ANOVA test for a-d, and using unpaired two-tailed Student’s t test for e-f.


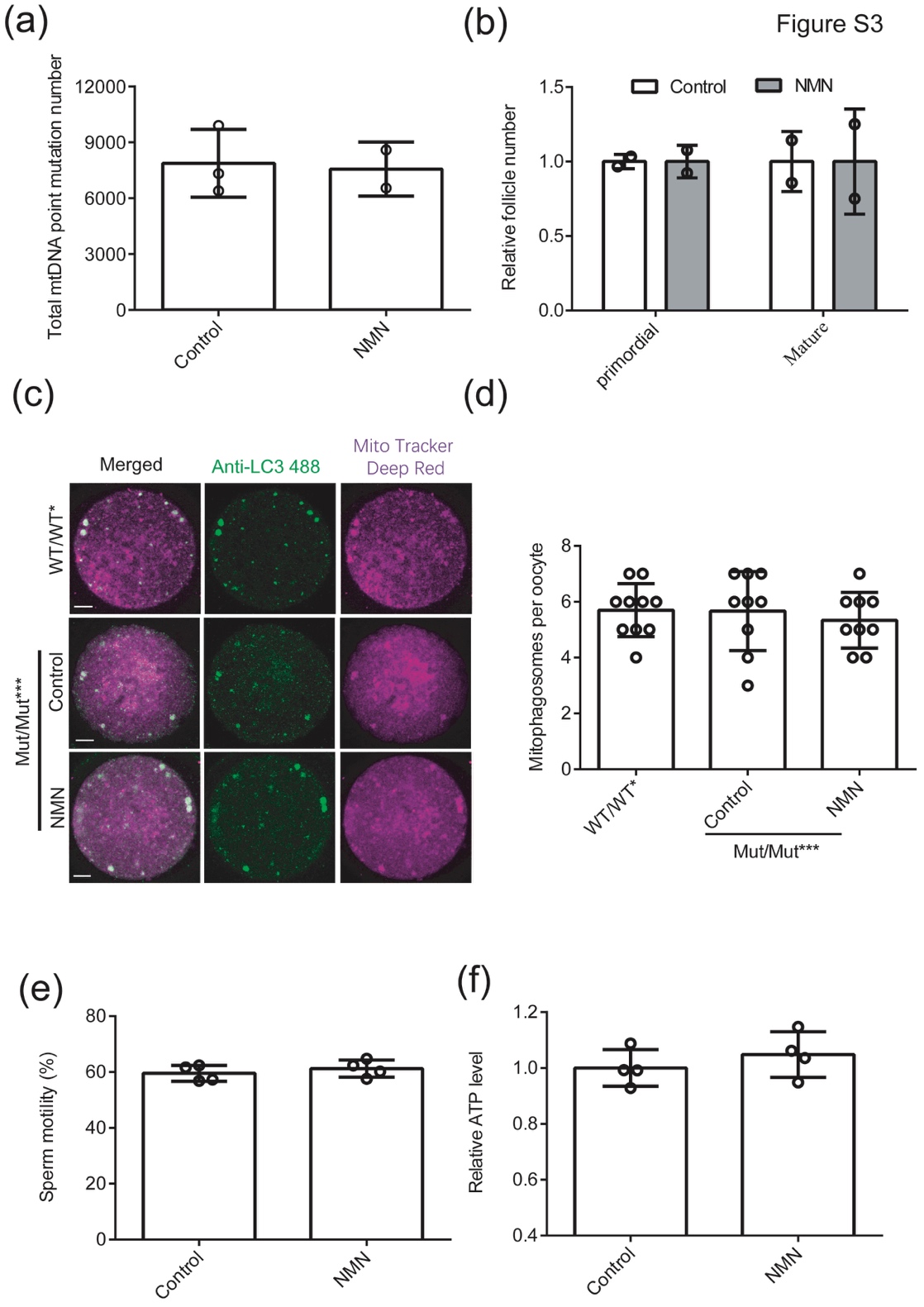


**Supplementary Figure 3, Related to Figure 5.** (A) The mtDNA point mutation number of the follicles of Mut/Mut*** female mice treated with NMN (n=2) or water control (n=3). (B) The ovarian primordial and mature follicles in Mut/Mut*** female mice treated with NMN (n=2) or water control (n=2). (C) The representative images of mitophagy in oocytes from Mut/Mut*** mice treated with NMN or water control, and WT/WT* mice (mitochondria are shown in pink, and LC3 in green; Scar bar, 10 μm) (D) Number of LC3 dots overlapping with Mito Tracker Deep Red from C (n=10 oocytes collected from 2 female mice for each genotype). Sperm motility (E) and relative ATP level of sperm (F) in Mut/Mut*** male mice treated with NMN (n=4) or water control (n=4). Error bars are SD.

Table S1. Primers for amplifying the whole mtDNA of human oocytes

| Region | name | Sequence (5’to 3’) |
| --- | --- | --- |
| 3561-9794 | 3561-FP | ATGAACCCCCCTCCCCATACCC |
|  | 9794-R1 | TGTTGAGCCGTAGATGCCGTCGGAAAT |
| 9795-14567 | 9795-F2 | TTTTTTGTAGCCACAGGCTTCCACGGACT |
|  | 14567-R2 | TGTGGTCGGGTGTGTTATTATTCT |
| 14562-139 | 14562-F3 | CCACACCGCTAACAATCAAT |
|  | 139-R3 | GAATCAAAGACAGATACTGCGACAT |
| 115-3560 | 115-F4 | ATGTCGCAGTATCTGTCTTTGATTC |
|  | 3560-RP | AGTAGAAGAGCGATGGTGAG |

Table S2. Primers for amplifying the whole mtDNA of mice sperm and follicles

| Region | | name | | | Sequence (5’ to 3’) |
| --- | --- | --- | --- | --- | --- |
| 1872-6222 | 1872-FP | | GGAATGCCTAAAGGAAAGATCCAAAAAGATAA | | |
|  | 6222-RP | | | CATCTAATCCTACTGTGAAT | |
| 6203-10627 | 6203-FP | | | ATTCACAGTAGGATTAGATG | |
|  | 10627-RP | | | AGGGTATAAAATAGGAAA | |
| 10622-1871 | 10622-F | | | TACCCTAATCGGTTCTATTCCACTGCTAATTG | |
|  | 1871-R | | | GGTGTTGGGTTAACAGAGAAGTTATAGGTGGA | |
